# Should We Consider the Cardiovascular System While Evaluating CKD-MBD?

**DOI:** 10.3390/toxins12030140

**Published:** 2020-02-25

**Authors:** Merita Rroji, Andreja Figurek, Goce Spasovski

**Affiliations:** 1 University Department of Nephrology, Faculty of Medicine, University of Medicine Tirana, Tirana 1001, Albania; 2 Institute of Anatomy, University of Zurich, Zurich 8057, Switzerland; andrejafigurek@yahoo.com; 3 University Department of Nephrology, Medical Faculty, University of Skopje, Skopje 1000, North Macedonia; spasovski.goce@gmail.com

**Keywords:** chronic kidney disease, uremic cardiopathy, left ventricular hypertrophy, phosphate, PTH, FGF23, klotho, sclerostin

## Abstract

Cardiovascular (CV) disease is highly prevalent in the population with chronic kidney disease (CKD), where the risk of CV death in early stages far exceeds the risk of progression to dialysis. The presence of chronic kidney disease-mineral and bone disorder (CKD-MBD) has shown a strong correlation with CV events and mortality. As a non-atheromatous process, it could be partially explained why standard CV disease-modifying drugs do not provide such an impact on CV mortality in CKD as observed in the general population. We summarize the potential association of CV comorbidities with the older (parathyroid hormone, phosphate) and newer (FGF23, Klotho, sclerostin) CKD-MBD biomarkers.

## 1. Introduction

Over the past 25 years, chronic kidney disease (CKD) has become an enormous public health issue with a high risk of morbidity and fatal outcome. Cardiovascular disease (CVD) is the most frequent (39%) cause of mortality in this population of end-stage renal disease (ESRD) [1], whereas the risk of CV mortality in early-stage CKD far exceeds the risk of progressing to dialysis [2]. Cardiovascular involvement is evident, initiates in the early stages of CKD (according to the K/DOQI CKD classification), being present in about 80% of prevalent hemodialysis patients [1]. CKD being recognized as an independent risk factor for CVD is a topic of debate on whether it should be recognized as a coronary disease risk equivalent, independent from the risk of diabetes and hypertension [1]. 

The complicated relationship between CVD and kidney disease reflects the interaction of traditional, non-traditional cardiovascular risk factors modified by CKD, and new CKD linked risk factors like uremic toxins, CKD-mineral and bone disorder (MBD), anemia, hypervolemia, oxidative stress, inflammation, insulin resistance, etc. [3,4]. Uremic toxins with presumed cardiovascular toxicity including FGF23 and protein-bound uremic toxins (PBUTs) like indoxyl sulfate, p-cresyl sulfate, start to accumulate in the body since early-stages of CKD, and elimination no longer relies on only renal replacement therapy. It is more than clear that CVD in CKD is an accelerated atherosclerosis.

Out of the five subtypes of cardiorenal syndromes classified so far, primary CKD leading to an impairment of cardiac function, can be established in the context of cardiorenal syndrome type 4 [5]. The interrelation between reduced renal function and altered cardiac remodeling in patients with CKD is termed uremic cardiomyopathy [6]. 

CKD-related cardiomyopathy has multifactorial pathophysiology. Here the effect of CKD-MBD has been already extended beyond the role in the skeleton. The pathogenesis of CKD-MBD has initially been described as a decrease in 1,25-dihydroxy vitamin D [1,25(OH)2 D3] levels leading to increased serum parathyroid hormone (PTH) level, following changes in calcium and phosphorus metabolism [7]. Vitamin D deficiency, together with secondary hyperparathyroidism (sHPTH) and hyperphosphatemia, was defined as the main factor influencing high cardiovascular risks in CKD patients [7]. The identification of new players such as FGF23, klotho [3], and sclerostin has changed what has been portrayed above because of their role not only in the sHPTH pathophysiology but also throughout their direct or indirect involvement in the uremic cardiovascular disease [7]. FGF23, klotho, Fetuin-A/Calciprotein particles, and sclerostin could be used among other old and relevant markers, as biomarkers for CV risk prediction in CKD [8].

We summarize here the potential association of those comorbidities with the older (parathyroid hormone, phosphate, Vit D deficiency) and newer (FGF23, Klotho, sclerostin) CKD-MBD biomarkers [2].

### 2. Role of Phosphate, Parathyroid Hormone and Vit D Deficiency in Uremic Cardiomyopathy

#### 2.1. Pathophysiology of Uremic Cardiomyopathy in CKD Patients

Uremic cardiomyopathy in patients with CKD or ESRD is a result of the volume and pressure overload, and the uremic state itself, including left ventricular hypertrophy (LVH), the diffuse interstitial fibrosis, and microvascular disease [3,5,6]. Histopathological examination of postmortem cardiac tissue samples in hemodialysis patients showed increased cardiomyocyte diameter, reduced capillary length density, and increased interstitial volume [9].

##### 2.1.1. Left Ventricular Cardiomyopathy

LV hypertrophy is the most frequent cardiac finding in dialysis patients, and it is almost universal [8]. The prevalence of LVH is estimated to be between 16% and 31% in individuals with GFR >30 mL/min; it rises to 60%–75% before renal replacement therapy initiation and increases up to 90% after the dialysis initiation [10]. It is related to chronic volume and pressure overload, neurohormonal activation, and uremic toxin accumulation [11]. The pathophysiological factors involved in LVH of CKD patients are (1) related to afterload, (2) related to preload, and (3) not related to afterload or preload [5,12,13,14]. The ones in the first group give a picture of an increase in systemic arterial resistance, elevated arterial blood pressure, and reduced large-vessel compliance [11,12,13,14] partially correlated to aortic ‘calcification’, which is specific in CKD patients. LV hypertrophy is a compensatory response that acts to maintain wall stress in the course of long-term loading conditions, where all these factors lead to myocardial cell thickening and concentric LV remodeling. Among the preload-related factors, the role of intravascular volume expansion (salt and fluid retention), secondary anemia, and the presence of arteriovenous fistulas which result in myocardial cell lengthening and eccentric or asymmetric LV remodeling need to be underlined. Both afterload and preload-related factors act with additive and/or synergistic effects. It is suggested that fluid overload and increased arterial stiffness play a role in LVH even before the start of dialysis therapy [15]. Arteriosclerosis, being a hallmark of arterial remodeling in ESRD, is characterized by diffuse calcification in combination with dilatation, and an increased wall thickness of the medial layer of the aorta and its main branches which drives increased arterial stiffness [11,16,17]. Here, LVH happens regardless of the effective control of hypertension. Blood pressure independent LVH also occurs in diabetics with known diabetic nephropathy [18].

Hypertrophied hearts have reduced coronary blood flow reserve and are at increased risk for myocardial ischemia [19]. The coexistence of left atrial enlargement is common, and atrial fibrillation occurs frequently. Eventually, continuing LV load can promote structural changes in the LV, apoptosis of cardiomyocytes, and triggers metabolic pathways able to increase the extracellular matrix production up to fibrosis [9,10,20,21]. 

##### 2.1.2. Interstitial Fibrosis

Diffuse interstitial cardiac fibrosis is reported in uremic patients and progresses with advancing of CKD [11,13,20,21,22]. Recently, it was nicely reported that in early-stage CKD patients, noninvasive imaging biomarkers of myocardial fibrosis do not change if renal function remains stable [22]. Fibrosis alters the architecture of myocardium promoting the progression of cardiac disease (progressive impairment in contractility, systolic and diastolic dysfunction, dilated cardiomyopathy, congestive heart failure) towards heart failure (HF) and increase the risk for sustained atrial and ventricular arrhythmias [9]. This may explain why CKD patients are at increased risk of sudden cardiac death (SCD) [23]. Recent studies have pointed out that not only CKD-MBD well-known biomarkers like phosphate, vit D, and PTH [3,5,7] but also novel and early ones like FGF23 are involved in the regulation, growth, and differentiation of cardiac myocytes being players in the pathogenesis of LVH [3,5,11,12].

##### 2.1.3. Microvascular Disease

The coronary microvascular function is not well studied in CKD. Based on one old report around 30% of dialysis patients with clinical angina have only moderate epicardial coronary artery disease (CAD) [24], possibly explained with endothelial dysfunction associated with microvascular disease [11,25]. The presence of structural and coronary functional changes contributes to myocardium-capillary mismatch which is not specific to uremia [9]. Under the condition of disbalance between high oxygen demand and a low oxygen supply microvascular coronary disease exposes cardiomyocytes to the risk of hypoxemia and beyond in possible ischemic myocardial injury at the microvascular level, which could be an explanation for persistently elevated serum troponin levels found in these patients [3,26].

Coronary artery calcification (CAC) as measured by computed tomography is noninvasive with excellent accuracy measurement of the burden of coronary atherosclerosis. CKD patients have higher CAC scores compared with age-matched controls without CKD, and those without baseline calcification present higher incidence rates of developing future de novo CAC [27]. Besides traditional factors, here, in particular, there are nontraditional risk factors such as hyperphosphatemia, calcium-phosphorus product, homocysteine, osteoprotegerin, and sclerostin which were independently related to the presence and high CAC scores [8,27,28].

#### 2.2. Role of Phosphate in Uremic Cardiomyopathy

Phosphate toxicity is a well described phenomenon in CKD [29,30]. Less than 1% of total phosphate is found in the blood and its balance was regulated by the interplay of bone, the parathyroid glands, intestines, and kidneys. The kidney is the principal organ regulating phosphate homeostasis. Following the loss of glomerular filtration rate (GFR), tubular phosphate reabsorption is significantly decreased by dual effect of compensatory increased concentration of two important hormones, the parathyroid hormone (PTH), and fibroblast growth factor 23 (FGF23). In addition, FGF23 suppresses the activation of vitamin D and acts to decrease parathyroid hormone synthesis and secretion being the major trigger in the path of CKD-MBD. FGF23 needs its cofactor klotho to ensure phosphate clearance [31]. Since the expression of Klotho declines in the kidney in the earlier stage CKD, FGF23 rises due to the resistance to FGF23 signaling in the kidney [31,32]. Although renal α-Klotho levels were significantly reduced and serum FGF23 levels were significantly elevated they can maintain serum phosphate within the normal range in early and intermediate stages of CKD. However, as CKD progresses, these defense mechanisms are ineffective, so phosphate retention may occur, and hyperphosphatemia develops.

Elevated serum phosphate has revealed as a non-traditional risk factor for cardiovascular events in CKD and partially explains the increased mortality risk in CKD [29,31,32]. 

The role of phosphate in vascular calcification has been the focus of intense investigation in the past decades. In elevated phosphate conditions, the biology of the arterial tunica media is found greatly altered; there is vascular smooth muscle cell (VSMC) transition to bone phenotype, apoptosis inactivation of local anti-calcification factors, and elastin degradation [33,34]. 

The PiT-1 phosphate transporter seems to be a key mediator in phosphate-induced VSMC, activating bone formation-related gene expression, osteochondrogenic differentiation, and was recently shown to be relevant in cell proliferation and embryonic development, referring more functions for this protein than previously thought [33,34,35]. Vascular mineralization, especially affecting the coronary artery, is strongly related to mortality of CKD patients independently from the established atherogenic markers. The rate of coronary artery mineralization in CKD patients undergoing hemodialysis treatment was reported to be five times higher than in the non-dialysis CKD patients and is associated with features of valvular calcifications sharing the same changes [16,31,36]. Moreover, valvular heart disease is one of the most common complications observed in patients with CKD [37,38] and hyperphosphatemia directly affects progression of valvular calcification.

The progression of valvular calcification leads to obstruction of left ventricular outflow and inflow from the left atrium to the left ventricle associated with hemodynamic changes resulting in very difficult clinical conditions [39].

Endothelial dysfunction is another early and crucial step in the development of cardiovascular disease apart from vascular calcification. Fewer reports have shown that phosphate level not in the physiologic range directly affects endothelial function and vascular remodeling [40,41]. Elevated phosphate level impairs endothelial function, hence diminishing microvascular function, angiogenic ability, and promoting endothelial stiffness [42].

Endothelial stiffness reflects changes in the structural and functional properties of the endothelium. These include cytoskeleton restructuring, successive mechano-signaling activity, intensified endothelial turnover (apoptosis), and diminished NO bioavailability [42].

High serum phosphate levels in HD patients were found to be independently associated with an increased number of endothelial microparticles (EMPs) and circulating (detached) endothelial cells [43]. These circulating submicron-sized membranous vesicles released by endothelium have a major biological role in the vascular injury; EMPs have been shown to act as primary and secondary messengers of vascular inflammation, thrombosis, vasomotor response, angiogenesis, and endothelial survival.

**Figure 1:**
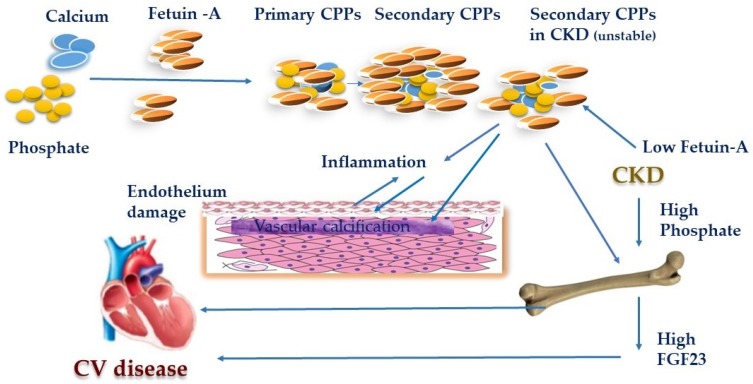
Role of Calciprotein particles in cardiovascular disease. In chronic kidney disease (CKD) patients, secondary Calciprotein particles (CPPs) have lower levels of calcification inhibitors including fetuin-A and were readily taken up by the vascular smooth muscle cells (VSMCs) inducing vascular calcification. Phosphate seems to be the driving force of CPP formation. Figure 1 shows the significance of CPPs, its contributions to bone and mineral metabolism, in an inflammatory response, and its role in the cardiovascular damage.

Phosphate is the major contributor to the level and biological activity of Calciprotein particles (CPPs) which are a new biological marker of CKD-MBD. Reports have shown that phosphate alone is not able to induce VSMCs mineralization, describing a synergistic action of both Ca and P in accelerated mineralization in vitro [16]. Insoluble CaP crystals generate when the concentration of calcium and phosphate exceeds the solubility limit. They can grow over time and finally precipitate as hydroxyapatite. The hepatic plasma protein fetuin-A (a natural calcification inhibitor) stabilizes colloidal protein–mineral complexes in the form of CPPs and mediates their clearance from the circulation. Primary CPPs, further, undergo topological rearrangement to find a more stable structure introduced as secondary CPPs [44]. The formation of CPP can be considered as a defense mechanism that prevents blood vessels from being occluded with insoluble CaP precipitates. The CPP level increases in the early stages of CKD, just before the rise of FGF23 and there are clinical findings that raise the hypothesis that CPPs might induce FGF23 [44,45]. In CKD patients, secondary CPPs have lower levels of calcification inhibitors including fetuin-A, and Gla-rich protein, readily taken up by the VSMCs inducing vascular calcification. While phosphate seems to be the driving force of CPP formation, his partner calcium seems to be a promoter of the inflammation-associated tissue damage forming a circle where increased mineralization triggers inflammation and vice-versa [44]. Recent studies have figured out the physiological and pathological significance of CPPs, its contributions to bone and mineral metabolism, and its role in tissue and organ impairments especially in cardiovascular damage and inflammatory responses [16,46,47] (Figure 1). Based on these findings secondary CPPs could be a new biomarker for the pathological condition of CKD-MBD [47]. More studies are required to further clarify the role of CPPs as an essential mediator of CV damage and as a potential therapeutic target in CKD patients [47]. Recently, Ciceri et al. reported that ferric citrate prevents high Pi-induced calcium deposition by preventing apoptosis. Apoptosis has been proposed to be one of the mechanisms that initiate the calcification process by forming a nidus for the deposition of calcium and Pi crystals. Even in the status where VSMCs are already transformed with a procalcified stimulus being present, reverting apoptosis and inducing autophagy presumably contribute to stopping calcium deposition [48]. 

Animal experimental data suggest that higher dietary phosphate engages multiple mechanisms involved in hypertension, including overactivation of the sympathetic nervous system, increased vascular stiffness, impaired endothelium-dependent vasodilation, together with an increased renal sodium absorption or renal injury [49]. 

On the other hand, there is limited evidence of a hyperphosphatemia-induced direct effect on cardiomyocytes. Dietary phosphate intake and hyperphosphatemia were frequently associated with abnormalities of the postcoronary arterial vessels in the myocardium and to interstitial fibrosis where hyperphosphatemia accelerate cardiac fibrosis as well as microvascular disease in experimental uremia [9,50]. In vitro studies showed that high Pi alone may not be able to generate cardiac hypertrophy but can initiate fibrosis [51]. Fibrosis, arising from non-myocytes and enhanced by cardiac myocytes, can promote increased wall stiffness and diastolic dysfunction. Moreover, fibrosis interrupts electrical signals, causing the tissue to be more arrhythmogenic [9,23]. Cardio markers and parameters of myocardial function, including Cardiac troponin T (cTnT), left ventricular max index (LVMi), left atrial dimensions (LAD), left ventricular end-systolic dimension (LVDs), left ventricular end-diastolic dimension (LVDd), interventricular septal thickness (IVST), and left ventricular posterior wall thickness (LVPWT), were reported consistently higher in a group of patients with higher serum phosphate (HSP) levels compared to those in the normal serum phosphate group (NSP) group, while left ventricular ejection fraction (LVEF) showed the opposite trend in a CKD cross-sectional study [52]. Furthermore, the lack of difference in mean arterial pressure (MAP) between the two groups suggested that cardiac remodeling including LVH and the declining LVEF might be associated with serum phosphate rather than hypertension and possibly this happens through triggering apoptosis of human cardiomyocytes. 

With respect to CV mortality, it is reported that risk assessment varied from 1.09–1.13 for phosphorus (every 1 mg/dL increase) to 1.13–1.28 for calcium (every 1 mg/dL increase) [53].

**Figure 2:**
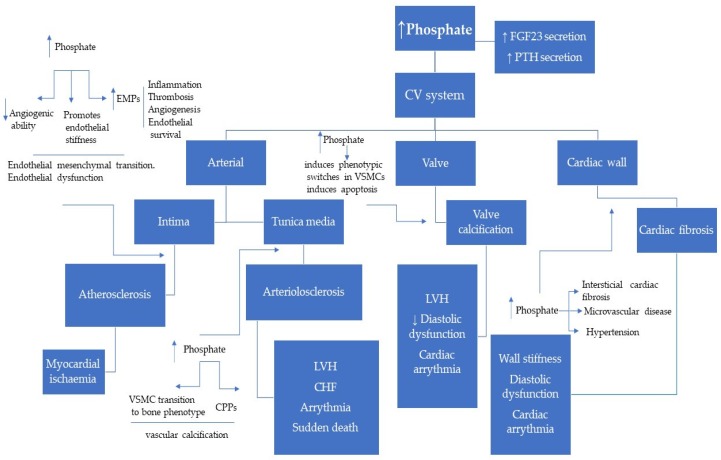
Pathophysiology of phosphate toxicity in the cardiovascular system. In CKD, higher serum phosphate levels are consistently linked with clinical and subclinical cardiovascular disease. Abbreviations: CPPs—Calciprotein particles; EMPs—endothelial microparticles; LVH—left ventricular hypertrophy; CHF—chronic heart failure; VSMCs—vascular smooth muscle cells; ↑ elevate; ↓ decrease.

In conclusion, enhanced phosphate has detrimental effects on the cardiovascular system seriously affecting patient outcomes (brief summary presented in Figure 2). Phosphate is toxic, impairs endothelial cells, promotes the formation of CPPs, induces VSMC transformation to osteogenic phenotype, and initiates cardiac fibrosis that leads to cardiac remodeling.

#### 2.3. Role of Parathyroid Hormone

Secondary hyperparathyroidism is a frequent complication of CKD characterized by an increase in PTH synthesis and secretion and by parathyroid gland hyperplasia. High levels of PTH have an impact on the cardiovascular system apart from the regulation of calcium and phosphate homeostasis [54]. Elevated PTH levels are a common finding in uremic patients which appears much earlier than hyperphosphatemia. PTH and FGF23 have both phosphaturic effects. The difference remains that only PTH has an impact on increased serum calcium. While PTH receptor (PTH1R), is present in bone and kidney, the klotho coreceptor is only expressed in the kidney [7,31]. In addition, PTH stimulates calcitriol synthesis that further contributes to increased serum calcium, whereas FGF23 has an opposite effect on vitamin D and calcium. In the physiologic state, FGF23 acts on the parathyroid gland by reducing gene expression and secretion while in the absence of Klotho, the parathyroid gland shows resistance to FGF23, so enhances PTH secretion.

Experimental data have shown that PTH may directly affect the myocardium although the effect of PTH on the CV system is still under study. 

PTH was shown to affect directly rat myocardial cells causing early death of cells by increasing calcium entry into the heart cells [55]. Calcium ions are crucial to myocardial excitation–contraction coupling and cardiac contraction and relaxation [56].

There are early reports by Amman et al. regarding the non-hemodynamic effect of PTH on cardiac fibrosis which was related to diastolic LV function [9]. An experimental rat model of CKD (5/6 nephrectomy) reported that continuous infusion of supraphysiological rates of synthetic PTH in animals with parathyroidectomy was associated with an extensive progression of VC—independently of serum Pi levels or the presence of uremia [57]. Moreover, the higher PTH levels have direct trophic effects on cardiomyocytes, interstitial fibroblasts, and smooth muscle cells of intramyocardial arterioles, promoting cardiac hypertrophy and fibrosis. PTH activates fibroblasts and regulates pro-fibrotic factors, such as aldosterone and angiotensin II (PTH stimulates aldosterone secretion by increasing the calcium concentration in the cells of the adrenal zona glomerulosa directly by binding to the PTH/PTH-rP receptor and indirectly by potentiating angiotensin 2 induced effects) [54,58]. Additionally, PTH potentially would activate protein kinase C, which further on activates other proteins, such as TGF-b, that in turn, promote the proliferation of fibroblasts, collagen synthesis, and fibrosis [59,60]. In vitro studies have found that PTH shows to have chronotropic, inotropic, as well as hypertrophic effects on cardiomyocytes [55] and based on research it was represented that there is a source for a direct role of PTH on cardiac electrophysiology outside of its effect on serum calcium [61].

Furthermore, ex-vivo experiments have shown the interaction between PTH and norepinephrine release in isolated human atria and renal cortex tissue through activation of the PTH1-receptor subtype. This effect would be an explanation for another potential underlying mechanism of the sympathetic overactivity and the associated cardiovascular mortality seen in patients with ESRD [62].

In hemodialysis patients, like in the rat model, the effect of PTH on the myocardium and cardiac fibrosis was well perceived. The hormone was shown to raise the beating rate of myocardial cells and induced their death after prolonged hormonal exposure; PTH stimulates the cyclic AMP production and impairs energy production, transfer, and utilization by myocardial cells [63] and myofibrillar activity of creatine kinase [64]. The presence of sHPTH has also been shown to correlate with enhanced myocardial calcium content and impaired ventricular systolic and diastolic function [65]. 

Despite a theoretical inverse association between plasma PTH concentration and left ventricular function, parathyroidectomy is not consistently associated with improvement in cardiac contractile function [66]. This suggests that changes induced by PTH could be irreversible in the case of long-standing severe hyperparathyroidism, or other factors contributing to myocardial dysfunction were more important than PTH excess or PTH interferes with the other risk factors of CVD. Despite a theoretical inverse relation between plasma PTH concentration and left ventricular function, parathyroidectomy is not consistently associated with improvement in cardiac contractile function [66]. This suggests in the case of long-standing severe hyperparathyroidism changes induced by PTH could be irreversible, or other factors with an impact on myocardial dysfunction are more important than PTH excess or PTH interferes with the other risk factors of CVD. Furthermore, the inconclusive results of the EVOLVE trial have been linked with this uncertainty since in intent-to-treat analysis a significant advantage of cinacalcet treatment over best presently available standard treatment in the combined primary endpoint (cardiovascular events plus death) was not shown, despite a marked decrease in serum PTH [67]. However, in the subanalysis, when it was adjusted for major confounders such as age and study drug discontinuation, the better control of hyperparathyroidism correlated with a significant advantage in hard outcomes. It was reported that PTH increases with age, weight, BMI, SBP, and LDL, all risk factors for CVD. Increased SBP would be a hemodynamic effect of PTH on cardiac remodeling [68,69]. Evidence suggests that PTH has vascular effects [68]. Here its potential effects on endothelial dysfunction, and increased serum levels of endothelin-1 and IL-6 could be mentioned. In addition, PTH may stimulate the vascular smooth muscle cells to produce factors including collagen and beta-1 integrin which could, in turn, remodel the peripheral vasculature. Another potential effect of PTH would be the increase of renin release and activation of the renin–angiotensin system, a process mediated by serum calcium and renal 1-alpha hydroxylase [69]. Aman et al. have underlined the effect of PTH as the major determining factor of coronary artery lesions, ranging from the discontinuity of the elastic lamina to the calcification of the medial layer, confirming the agreeable action of PTH [70].

In conclusion, there are clinical and experimental reports which support the hypothesis that PTH behaves as a systemic uremic toxin, with direct and indirect effects on uremic cardiomyopathy. PTH acts through four major cardiovascular effects; contractile disturbance, cardiomyocyte hypertrophy, cardiac interstitial fibrotic, and vasodilator effect. Severe sHPTH is an important threat to CKD patient outcomes affecting CV morbidity and mortality and remains an important therapeutic target to prevent bone and CV complications in such patients.

#### 2.4. Role of Vitamin D

During the last decades, the role of Vit D on CV events has triggered a lot of studies where observational studies (OS) have reported an association of vitamin D deficiency with cardiovascular disease, including carotid intima-media thickness, peripheral vascular disease, and cardiovascular death. Vitamin D supplementation diminishes levels of inflammatory markers and lipids (particularly triglycerides), improves endothelial function (as measured by brachial artery flow-mediated dilatation) and blood pressure (BP) control in the general population with or without vitamin D deficiency [71]. Besides, nephrologists have supported supplementation with 1,25-dihydroxy vitamin D in patients with ESRD since the inactivation of Vit D with the progression of CKD was known. If not managed on time, 1,25(OH)2D deficiency might promote the classic view of mineral and bone disorders (MBDs) such as secondary hyperparathyroidism and osteitis fibrosa cystica. These abnormalities together with endothelial dysfunction and vascular changes from the early stages of CKD [72], results in further vascular calcification and arterial stiffness [73]. Vitamin D has been shown to have anti-inflammatory and anti-oxidative properties and additionally downregulates the expression of renin, correlating with an increased prevalence of hypertension, heart failure, CV events, and a higher CV mortality rate in CKD [74,75,76]. 

In vitro data have shown a direct effect of vitamin D on endothelial function, related to decreased oxidative stress and increased levels of endothelial nitric oxide synthase (eNOS). These findings are supported by the promising results of a few randomized clinical trials which represented beneficial effects of nutritional vitamin D supplementation or paracalcitriol on endothelial function (brachial artery flow-mediated dilatation) in CKD stage 3–4 [77,78]. Other positive effects on Vit D supplementation were noticed on inflammation markers, intracellular cell adhesion molecule, vascular cell adhesion molecule, E-selectin parathyroid hormone, and arterial stiffness [79].

A recent meta-analysis supports the positive effect of vitamin D intervention on endothelial function mainly in younger patients, apparently due to an earlier diagnosis, where vascular remodeling has not yet been established. Limitations of this meta-analysis were the small number of studies included, and the short duration of intervention suggesting a need for larger and longer studies on this topic, with sufficient power to assess hard endpoints [80]. The controversies remain also on the impact of Vitamin D on cardiac structure and function.

Experimental studies through a specially engineered mouse model have shown that targeted deletion of the vitamin D receptor gene increased cardiomyocyte size and LV weight without fibrosis [81]. Similarly, an association between vitamin D deficiency and increased myocardial collagen content, impairment of cardiac contractile function, and increased cardiac mass was reported previously [82,83]. On the other hand, beneficial effect of treatment with activated vitamin D on attenuation of myocardial hypertrophy [84] and prevention of heart failure [85] in experimental models were not supported neither by Primo and Opera trials, which showed that 48 or 52 weeks of treatment with paricalcitol, respectively, at a dose that adequately controls secondary hyperparathyroidism did not regress LV hypertrophy or improve LV systolic and diastolic dysfunction in CKD stage 3–5. Moreover, the promising effect of lowering CV-related hospitalizations needs further confirmation [86,87]. 

In addition, based on the data of the recent meta-analysis including 38 studies involving 223, 429 patients (17 RCTs, *n* = 1819 and 21 OSs, *n* = 221,610) it could be concluded that that the existing RCTs that used the intention-to-treat principle do not provide an adequate or conclusive evidence that Vit D supplementation affects the mortality of patients with CKD while in observational studies Vit D treatment was significantly correlated with a 38% reduction in all-cause mortality and 45% reduction in CV mortality. The different findings between the RCTs and OSs demonstrate that confidence on neither should be absolute and the conclusion was that large-size RCTs with a proper dose and sufficient treatment time, in the true vitamin D-deficient patients with CKD are needed in the future to assess, prospectively, any potential differences in survival [88].

## 3. Importance of New CKD-MBD Biomarkers in Early Cardiovascular Risk Assessment

Considering significant CV risk and mortality in patients with CKD, there is a growing attempt to find a reliable biomarker that would timely detect not only kidney disease but also define patients under higher risk to reduce CV mortality.

Compared to the “older” CKD-MBD biomarkers and already established in clinical routine, phosphate and PTH, which however display increased levels when CKD is already advanced, newer biomarkers, FGF23, Klotho, and sclerostin, give a bit more hope as there is growing evidence suggesting that their disturbed serum levels can detect initial CKD (Table 1).

### 3.1. Role of FGF23 

FGF23, a 32 kDa glycoprotein, has been defined as a phosphaturic hormone produced by osteocytes and osteoblasts [89]. In the physiological state, its main role is to maintain normal phosphate levels in the blood through downregulation of sodium-phosphate (NaPi) cotransporters in kidney proximal tubule and, thus, reducing the phosphate reabsorption in the kidney [89]. In addition, FGF23 downregulates 1-α-hydroxylase in proximal tubules, the enzyme responsible for converting 25-OH-vitamin D into his active form, 1,25(OH)2-vitamin D [89]. In this way, FGF23 regulates phosphate levels both directly, through NaPi cotransporters, and indirectly, through vitamin D metabolism and phosphate absorption in the gut.

FGF23 acts by binding with the transmembrane protein, α-klotho, which is expressed mainly in kidney proximal and distal convoluted tubule, parathyroid and pituitary glands, but also in other organs [90,91]. As FGF23 suppresses α-klotho expression in the kidney, it may decrease levels of secreted klotho in the circulation [90,92].

Studies performed so far confirmed that patients with CKD have increased FGF23 levels even from the early stages of the disease [93,94]. As high mortality in CKD patients is well known, the role of FGF23 in CV mortality was intensively investigated, both in experimental and clinical settings. A recent meta-analysis concluded that elevated FGF23 levels are positively associated with CV events and all-cause mortality in HD patients [95]. Data on repeated measurements of FGF23 levels in patients with CKD may identify subpopulation of patients that have higher mortality risk, as it was shown that those patients with slower rise in FGF23 levels in the course of five years have five times higher risk of death and those with rapid rise in FGF23 levels have 15 times higher risk of death compared to the patients with stabile FGF23 levels [96]. These data indicate that FGF23 acts as a toxin in developed CKD-MBD. Most of the studies investigating the association of FGF23 and mortality in CKD patients analyzed the presence of cardiac hypertrophy, known to be very common in CKD, and activation of the renin–angiotensin–aldosterone (RAAS) system. In patients with diabetic nephropathy and early CKD (stages 2 and 3), lower plasmatic Klotho and higher FGF23 levels were associated with a higher risk of concentric hypertrophy, and, thus, higher cardiovascular hospitalization [97]. It was shown that FGF23 stimulates the renin–angiotensin system by suppressing the expression of angiotensin-converting enzyme-2 (ACE2) in the kidney [98]. The study, which included both in vitro investigation of cardiac fibroblasts and myocytes and myocardial autopsy samples of patients with end-stage CKD, demonstrated that RAAS activation is responsible for the induction of FGF23 expression in cardiac myocytes and stimulation of pro-fibrotic crosstalk between cardiac myocytes and fibroblasts [99]. Besides, FGF23 also increases the production of transforming growth factor-β (TGF-β), lipocalin-2, and tumor necrosis factor-α (TNF-α), which are well known inflammatory markers [98]. 

Anemia is an important CKD complication that contributes to higher CV risk and mortality. It is important to underline that FGF23 also contributes to renal anemia development and inhibition of FGF23 signaling may decrease erythroid cell apoptosis, attenuate inflammation, and result in increased serum iron and ferritin levels [100]. Hence, it may be concluded that FGF23 increases CV risk either directly (by action on heart) and/or indirectly (RAAS activation, contribution to renal anemia, and inflammation), and also stimulates other pathophysiological factors that contribute to further disease progression. Regarding the relation to LVH, experimental data indicate that FGF23 can exert its action even if α-klotho is not present and to induce hypertrophy of cardiac myocytes [101]. Indeed, it has been shown that FGF23 directly induces LVH by activation of fibroblast growth factor receptor-4 (FGFR-4) in the absence of membrane α-klotho and that administration of soluble klotho attenuates hypertrophy in mice [102]. LVH, on the other hand, is shown to be associated with an increase in both myocardial and serum intact FGF23 [103]. 

Clinical data suggest the association of FGF23 levels and increased CV risk throughout the CKD stages. FGF23 is shown to be associated with increased risk of CV events and mortality in diabetic patients even with normal or mildly impaired kidney function [104]. Furthermore, FGF23 levels correlated positively with LVH and negatively to left ventricular ejection fraction in patients on hemodialysis, in whom FGF23 was shown to be an independent predictor of overall mortality [105]. 

Some authors pointed that predictive potential of FGF23 of CV mortality is more emphasized in patients in intermediate eGFR tercile (with mean value of 60 mL/min) [106].

These clinical data strongly support the role of FGF23 as direct cardiac toxin, which causes hypertrophy of cardiomyocytes that are exposed to less blood supply in the further course of the disease. Apart from the association with CV risk and mortality, the relationship of FGF23 with overall mortality can be explained through the stimulation of other pathways (inflammation for instance) that lead to CKD progression and mortality. 

Experimental data, on the other hand, indicate that the progression of LVH in CKD could be ameliorated. It is important to note that specific blockade of FGFR4, as shown in 5/6 nephrectomy rat model, attenuates LVH [107]. Moreover, experimental data in uremic rats indicated that vitamin D treatment reduced LVH, FGFR-4 expression, and calcineurin/nuclear factor of activated T cells (NFAT) signaling activation, and, therefore, showing calcitriol cardioprotective effects [108]. Encouraging experimental data also indicate that early administration of ferric citrate slows CKD progression, lowers FGF23 levels, and improves cardiac function and survival [109]. Hence, LVH can be treated in CKD and CV risk can be reduced, either by lowering FGF23 levels or by inhibiting its effect on the FGFR-4. To conclude, FGF23 acts as a toxin in CKD and has an important role in CKD-MBD development and, most importantly, is associated with increased CV risk in CKD patients. Therapeutic strategies to lower FGF23 serum levels and/or to inhibit its action on FGFR-4 might be beneficial for the CV and overall outcome improvement.

Early diagnosis of CKD-MBD is an appropriate time for prevention of CKD complications and reduction of CV risk. Monitoring FGF-23 levels could detect patients with higher CV risk and suggests more regular visits at nephrology departments.

### 3.2. Role of Klotho

In close relation to FGF23 levels elevation, it is known that patients with CKD are in klotho-deficiency, which, according to the existing knowledge, contributes to high CV mortality among CKD patients. Decreased soluble klotho levels in the circulation could be detected very early in CKD, from stage 2, and in urine even from CKD stage 1 [110].

On the cellular level, it has been shown that circulating klotho has a cardioprotective effect by downregulation of TRPC6 channels in heart as an antagonist of the Wnt/b-catenin pathway [111]. Klotho-deficient CKD mice had more pronounced cardiac hypertrophy than wild-type CKD mice and even after normalization of serum phosphate and FGF23 levels, cardiac hypertrophy was not improved, meaning that klotho-deficiency is an important cause of cardiac hypertrophy in CKD, independently of FGF23 and phosphate [112]. Klotho deficiency in CKD results not only in cardiac hypertrophy but is involved in cardiac fibrosis development. It has been shown that endogenous klotho is expressed both by human cardiomyocytes (HCMs) and cardiac fibroblasts (HCFs) and that uremic serum or TGF-β1 suppressed klotho expression by HCMs [113]. Klotho upregulation inhibits TGF-β1-induced fibrosis and pathogenic Wnt/ β-catenin signaling in HCMs [113].

Clinical studies also support the cardioprotective role of klotho. In patients with CKD 3 stage, a change in FGF23/klotho ratio correlated with the changes in left ventricular mass [114]. In hemodialysis patients, low klotho levels were associated with CV events, independently from other CKD-MBD factors [115]. Analysis of the LURIC (Ludwigshafen Risk and Cardiovascular Health) study did not show any additional predictive power of CV and mortality risk in patients with normal kidney function [116]. On the contrary, in patients with CKD, as presented by the KNOW-CKD study, serum klotho was shown to be an independent biomarker of a left ventricular mass index, but not of arterial stiffness [117]. 

Klotho deficiency also contributes indirectly to increased CV risk in CKD. Known to be expressed in the vasculature, klotho deficiency is involved in VC and endothelial dysfunction development [118] and, therefore, contributes to increased arterial stiffness and pressure overload. 

Experimental data indicate that calcified human aortic valves have lower klotho levels and that treatment with recombinant klotho reduces high phosphate-induced osteogenic activity in human aortic valve interstitial cells [119]. Another study confirmed that klotho administration attenuated high-phosphate induced renal and cardiac fibrosis and improved both renal and cardiac function in the absence of previous kidney disease [120]. Taken together, experimental data encourage that treatment of klotho deficiency in CKD may have a beneficial effect on heart disease in CKD. 

Whereas klotho did not predict CV events (death, atherosclerotic events, and decompensated heart failure) in patients CKD stages 2–4, FGF23, on the other hand, was significantly associated with future decompensated heart failure [121]. 

Bearing in mind klotho/FGF23 axis disturbance, the klotho deficiency, and high FGF23 levels, in patients with CKD, it has been suggested that the klotho/FGF23 axis could be not only diagnostic and prognostic biomarkers of CKD and CV disease but could be treatment targets as they contribute to the CKD progression and development of CV disease as complication [122].

### 3.3. Role of Sclerostin 

Sclerostin, a protein produced by osteocytes, and coded by the SOST gene on chromosome 17q12-q21, is an inhibitor of wingless-type mouse mammary tumor virus integration site (Wnt) pathway in osteoblasts, which is responsible for osteoblastogenesis [123]. In this way, sclerostin inhibits bone formation in a healthy state. Although previously described to be secreted as 27 kDa monomer only by osteocytes [124,125], later research pointed to the secretion also by other cells (osteoblasts, osteoclasts, chondrocytes, cementocytes) [126,127]. Interestingly, the SOST gene is found to be also expressed in other tissues and organs and, besides bone, primarily in heart, lung, aorta, and kidney [128,129]. Based on these data, sclerostin was no longer considered to be a bone-specific protein and marker of bone turnover, but the topic of further research aiming to understand its role in extraosseal tissues and organs. Unfortunately, the exact nature of sclerostin in those are not fully understood, neither in health, nor in a disease. Some of the limiting factors are the weak association between protein expression in the tissue and mRNA levels and different nature of sclerostin in different parts of the same tissue [130]. 

Clinical data on the association of sclerostin with CV risk and mortality are not very clear. It is known that patients with CKD have increased serum sclerostin levels already from the initial stages [131]. As the SOST gene is present in the heart and vascular tissue, the potential association of serum sclerostin with increased CV risk in CKD patients has also been investigated and is still an important topic in experimental and clinical research. However, compared to the studies investigating the association of FGF23 and klotho with LVH, most studies linked sclerostin with the presence of atherosclerosis and VC in CKD. Studies investigating the heart in CKD referred to the relationship between sclerostin and valvular calcification. In addition, sclerostin may exert an indirect effect on heart disease in CKD, by taking part in VC development and, hence, through increased peripheral vascular resistance and heart failure. 

Elevated serum sclerostin levels were seen in patients with aortic valve calcification with increased upregulation of sclerostin mRNA [132]. Sclerostin is shown to be an independent risk factor for heart valve calcification in patients with CKD stages 3–5 and is increased in serum before the increase in serum phosphate and PTH is seen [133]. In addition, in patients with CKD stages 2–5, serum sclerostin was reported to be associated with inflammation markers, phosphate, FGF23, indoxylsulphate and p-cresyl sulphate, β2-microglobulin, and arterial stiffness [134], emphasizing its role in CKD-MBD development.

High sclerostin serum levels (>200 pg/mL) were reported to be associated with increased carotid-femoral pulse wave velocity (>9.5 m/s) in HD patients [135]. Although during 2-year follow-up HD patients who died had higher sclerostin levels, sclerostin did not predict survival [136]. Similarly, to this study, it was reported that higher CV risk in HD patients was associated with sclerostin values above the median (>84pmol/L) during the five-year follow up period [137]. 

Recent experimental data suggest a positive correlation between the presence of VC in CKD rats and vascular Wnt3a and β-catenin expression together with blood pressure variability, but no association with sclerostin was seen [138]. In CKD patients, sclerostin was positively associated with VC (coronary arteries and thoracic aorta, but not with those at the aortic or mitral valves and it did not predict cardiovascular events) [139]. Meta-analysis performed by Kanbay et al. showed that serum sclerostin was not associated with all-cause and CV mortality [140]. Previously, it has been shown that serum sclerostin values were associated with fatal and nonfatal CV events in non-dialysis CKD patients [141]. On the other hand, the NECOSAD study indicated that incident dialysis patients with higher sclerostin level had better CV survival [142]. 

Up to now, there are some data suggesting the association of serum sclerostin with vascular and valvular calcification in CKD patients and the number of studies is very scarce with conflicting results. On the other hand, data on the potential relationship of sclerostin with uremic cardiomyopathy are lacking. Taken together, clinical studies on the role of sclerostin in CKD report inconclusive data and the exact role of sclerostin in CKD-MBD and CV risk is yet not clear with a need for further investigation. 

At present, it cannot be clearly stated whether serum sclerostin turns into a toxin in CKD and increases CV risk and mortality, or if it is only a marker of disturbed bone and (cardio)vascular and valvular metabolism. The critical point here is the ability to confirm the origin of high serum sclerostin levels and then to explain the reason for such increased values. Similarly, CKD-MBD treatment for reducing sclerostin levels is a double-edged sword. Although it has been shown that the application of anti-sclerostin antibodies improves bone and mineral density and reduces fracture risk in osteoporosis [143], there is also important data indicating that such treatment can increase CV risk in patients with primary osteoporosis [144]. 

Nevertheless, new studies on this topic should reveal the real physiological and pathophysiological roles of sclerostin in heart and vascular disease in patients with CKD and will direct future therapeutic strategies.

### 3.4. Role of OPG-RANK-RANKL System in CKD-MBD

**Figure 3:**
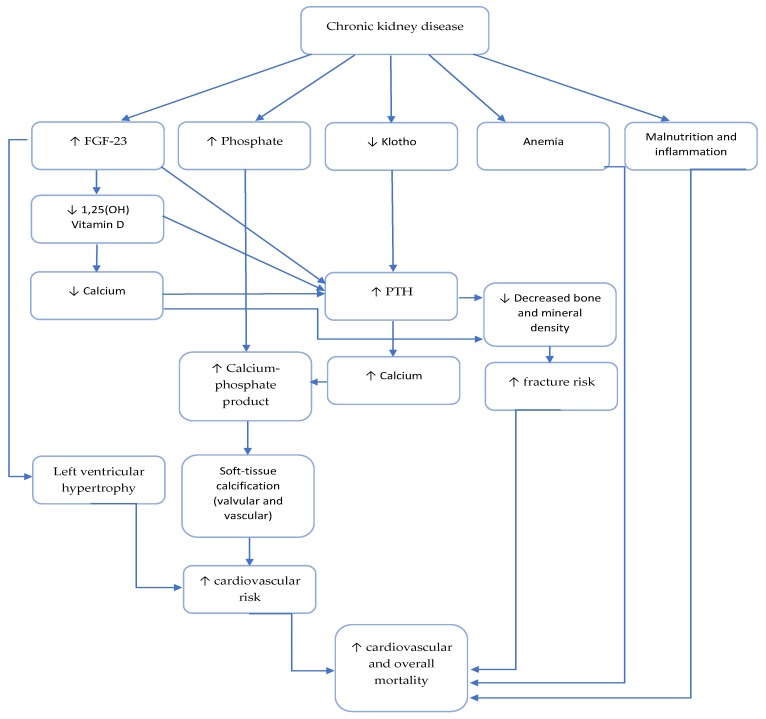
Mechanism of increased mortality in patients with chronic kidney disease. ↑ increases/increased; ↓ decreases/decreased.

Bone disease is an important component of CKD-MBD, that is linked to vascular disease and described as a calcification paradox [145] (depicted in Figure 3).

The disturbed OPG-RANK-RANKL pathway might be one of the contributors to bone disease and VC development in CKD. In physiological conditions, osteoprotegerin (OPG) is a protein which inhibits activation and differentiation of osteoclasts by blocking the binding of receptor activator of nuclear factor kappa–B ligand (RANKL) to RANK expressed on osteoclast precursors [145]. It has been shown that osteoprotegerin is produced by the arterial wall and other tissues [146]. 

Experimental data indicate that OPG knockout in mice is responsible for osteoporosis and VC development [147]. Moreover, OPG knockout mice displays higher RANKL and RANK levels, as well as OPG downregulation detected in calcified human arteries [148,149]. An important mediator of the opposite OPG-RANK-RANKL system regulation in bone and vasculature might be TGF-β, as it increases the OPG/RANKL ratio in bone and decreases in vasculature, disabling the VC inhibition by OPG [145]. Clinical data showed that coronary artery calcification score correlated positively with serum osteoprotegerin and negatively with RANKL, and serum osteoprotegerin correlated positively with the progression of coronary artery calcification score in hemodialysis patients [150]. 

Nevertheless, the calcification paradox seems to be very complex and most likely disturbs several pathways deserving more detailed experimental and clinical explanation.

## 4. Conclusions

Although the management of CKD patients was significantly improved, we are still faced with a high rate of CV mortality. In this review, we tried to go from each of the candidate mineral disorder to the CV abnormalities (summarized in Table 2). The risks of each mineral disorder from the oldest to the newest one varied with each kind of cardiac abnormality, which means that it is a significant challenge to prevent all cardiac abnormalities, even if CKD-MBD control has been guided in strict compliance with the guidelines. Therefore, we do have CKD-MBD markers acting as toxins: phosphate, PTH, and FGF23, as present important targets for treatment. On the other side, cardioprotective CKD-MBD markers such as vitamin D and klotho could be additional and very helpful points to treat. Finally, the newest CKD-MBD biomarker sclerostin, that interplays in CKD-MBD developing pathways, is still debatable concerning its protective role or acting as a toxin and consequently increasing CV risk development.

Diagnosis of CKD-MBD in the early development of CKD (stages 1 and 2) would be of great importance in preventing CKD progression, its complications, and would improve patients’ survival and quality of life.

Focusing on such toxins and/or their relevant mediators at early CKD stages might help to interfere over time with the vicious cycle of the cardio–renal connection, and improve the outcome of patients. Further clinical studies exploring the beneficial influence of therapy in CKD (vitamin D, iron replacement, anemia treatment, etc.) and the association to FGF-23 and sclerostin levels with the cardiovascular outcome, would be of great help in understanding the complex pathophysiological mechanism of CKD-MBD. 

## Figures and Tables

**Table 1:** The importance of the FGF-23–klotho–sclerostin axis in left ventricular hypertrophy in CKD.

FGF-23–Klotho–Sclerostin Axis	Cellular Level	Tissue Level	Circulation	Clinical Observation	Therapeutic Potential
FGF-23	FGF23 directly induces LVH by binding to the FGFR-4 in cardiomyocytes RAAS activation induces FGF23 expression in cardiac myocytes and stimulates pro-fibrotic crosstalk between cardiac myocytes and fibroblasts	FGF23 increases production of TGF-β, lipocalin-2, and TNF-α, and thus promoting the inflammation process	LVH is shown to be associated with an increase in both myocardial and serum intact FGF23 FGF23 contributes to renal anemia development -> contribution to LVH aggravation	FGF23 levels correlate positively with LVH and negatively to left ventricular ejection fraction in patients on hemodialysis	Vitamin D treatment reduces LVH Ferric citrate lowers FGF23 levels and improves cardiac function and patient survival
Klotho	Cardioprotective effect by downregulation of TRPC6 channels in cardiomyocytes, important for angiotensin II-induced hypertrophy signaling Klotho upregulation inhibits TGF-β1-induced fibrosis and pathogenic Wnt/ β-catenin signaling in cardiomyocytes	Cardiomyocytes and cardiac fibroblasts express klotho	Uremic serum or TGF-β1 suppressed klotho expression by cardiomyocytes	FGF23/klotho ratio correlates with changes in left ventricular mass Low klotho levels are associated with CV events Serum klotho is an independent biomarker of a left ventricular mass index	Klotho administration attenuates high-phosphate induced renal and cardiac fibrosis and improved both renal and cardiac function
Sclerostin	Lacking data about the association with LVH	Lacking data	Lacking data	Elevated serum sclerostin levels in patients with aortic valve calcification with increased upregulation of sclerostin mRNA	Not yet clear whether therapeutic decrease of sclerostin levels is beneficial or deleterious for CV outcome

Abbreviations: LVH—left ventricular hypertrophy; RAAS—renin–angiotensin–aldosterone system; TRPC6—transient receptor potential canonical type 6; TGF-β—transforming growth factor β; TNF-α—tumor necrosis factor α.

**Table 2:** CKD-mineral and bone disorder (MBD) biomarkers, role in bone metabolism and the cardiovascular system.

CKD-MBD Biomarkers	Role in Bone Metabolism	Vascular Calcification	Uremic Cardiomyopathy
**Phosphate**	Major trigger in CKD-MBD ↑P →↑PTH→ ↑Vit D →↑Ca ↑P →↑FGF23→↓Vit D→↓ Ca	Promotes VC Impairs endothelial function	Cardiac fibrosis
**PTH**	Key mediator of bone turnover Regulates P and Ca homeostasis	Complex paracrine and systemic effectPromotes VC Impairs endothelial function	Cardiac electrophysiology Cardiomyocyte hypertrophy Cardiac interstitial fibrosis
**Vit D**	Key role in Ca, P homeostasis Depletion promote sHPTH and osteitis fibrosis cystica	Biphasic curve of Vit D on calcification	Increases collagen ↓Vit D→ impairs contractile function Increases cardiac mass
**Klotho**	Acts as a Wnt-inhibitor Modify bone metabolism	Inhibitor of VC Klotho deficiency→ impair endothelial function	Klotho deficiency→ LVH Cardiac fibrosis
**FGF23**	Posphaturic hormone acts through α-klotho	Is not clear if it has a direct effect on VC	Concentric hypertrophy
**Sclerostin**	Inhibits bone turnover	Marker of vascular calcification	There are no conclusive data

Abbreviations: VC—vascular calcification; P—Phosphate; LVH—left ventricular hypertrophy; sHPTH—secondary hyperparathyroidism. → - brings to; ↓ decrease;↑increase.
